# Anti-phenolic glycolipid antibodies in *Mycobacterium bovis* infected cattle

**DOI:** 10.1016/j.onehlt.2025.100982

**Published:** 2025-01-28

**Authors:** Zijie Zhou, Anouk van Hooij, J. Hessel M. van Dijk, Nina Musch, Louise Pierneef, Hamza Khalid, Kees Franken, Thomas Holder, Neil Watt, Anita L. Michel, Jeroen D.C. Codée, Martin Vordermeier, Paul L.A.M. Corstjens, Elisabeth M.D.L. van der Heijden, Jayne C. Hope, Annemieke Geluk

**Affiliations:** a Department of Infectious Diseases, Leiden University Medical Center, Leiden, the Netherlands; b Department of Bio-Organic Synthesis, Leiden Institute of Chemistry, Leiden University, Leiden, the Netherlands; c Division of Immunology, The Roslin Institute, University of Edinburgh, Easter Bush Campus, Midlothian, EH25 9RG, United Kingdom; d Department of Bacteriology, Animal and Plant Health Agency, Woodham, New Haw, KT15 3NB, United Kingdom; e MV Diagnostics Ltd, Roslin Innovation Centre, University of Edinburgh, Easter Bush Campus, Midlothian, EHG25 9RG, United Kingdom; f Department of Veterinary Tropical Disease, Faculty of Veterinary Science, University of Pretoria, Onderstepoort, South Africa; g Department of Cell and Chemical Biology, Leiden University Medical Center, Leiden, the Netherlands; h Department of Biomolecular Health Sciences, Division of Infectious Diseases and Immunology, Faculty of Veterinary Medicine, Utrecht University, Utrecht, the Netherlands

**Keywords:** Antibodieshh, Bovine, Diagnosis, DIVA, *M. bovis*, Phenolic glycolipid, TB

## Abstract

*Mycobacterium bovis*, the causative agent of bovine tuberculosis (bTB), causes significant financial losses in the agricultural industry. Additionally, *M. bovis* transmission from animals to humans can result in zoonotic TB, especially in low- and middle-income countries (LMICs), highlighting the need to enhance One Health surveillance to mitigate this threat.

Antibodies directed against a major mycobacterial cell wall component of *M. leprae*, phenolic glycolipid-I (PGL-I), have shown excellent performance in identifying *M. leprae* infection in humans and animals. In this study, we therefore investigated whether antibodies against *M. bovis* PGL similarly represent a useful biomarker for *M. bovis* infection in cattle.

Comparing sera from naturally *M. bovis*-infected and the single intradermal comparative cervical tuberculin test (SICCT)-negative cattle, we assessed the potential of *M. bovis* PGL antibodies to identify this mycobacterial infection. Our results show that serum levels of anti-*M. bovis* PGL IgG and -IgM in *M. bovis*-infected cattle were significantly higher than in the SICCT-negative cattle. The sensitivity for anti-*M. bovis* PGL IgM in infected animals was, however, moderate (44.9 %) and the false-positive rate was 6.3 % in SICCT-negative cattle. Notably, vaccination with BCG- or heat-killed *M. bovis* did not affect serum levels of anti-*M. bovis* PGL IgM in cattle. Moreover, none of the 57 anti-*M. bovis* PGL-seropositive cattle tested positive in the anti-*M. leprae* PGL-I assay. This study shows for the first time that anti *M. bovis* PGL antibodies can be detected in infected cattle: anti-*M. bovis* PGL IgM is a highly specific, but moderately sensitive biomarker for *M. bovis* infection in cattle, showing potential for differentiate infected from vaccinated animals (DIVA). It could be a valuable component in a multi-biomarker approach for diagnosing bTB.

## Highlights

• *M. bovis* infection in cattle induces limited anti-*M. bovis* PGL serum antibodies.
• Anti-*M. bovis* PGL IgM is highly specific for *M. bovis* infection.
• Anti-*M. bovis* PGL IgM moderately improves diagnostics based on recombinant proteins.
• Anti-*M. bovis* PGL IgM supports differentiating infected from vaccinated animals (DIVA).
• Anti-*M. bovis* PGL-seropositive cattle do not test positive for anti-*M. leprae* PGL-I antibodies.

## 1 Introduction

*Mycobacterium bovis* is the primary causative agent of bovine tuberculosis (bTB) and can give rise to chronic infections, particularly in cattle. Globally, 50 million cattle are estimated to be infected with annual economic losses amounting to at least 3 billion US dollars [1]. In addition, *M. bovis* can cause zoonotic TB in humans: in 2019, an estimated 140,000 new cases and 11,400 deaths occurred due to *M. bovis* related TB in humans [2]. These numbers may be underestimated due to the lack of surveillance data, especially in low- and middle-income countries (LMICs) [3]. Early detection of *M. bovis* infection in cattle can prevent (zoonotic) transmission and strengthen the One Health approach to achieve the goals of the End TB Strategy of the World Health Organization (WHO) [4,5]. Reducing transmission of *M. bovis* between animals (including to and from wildlife species) would also have significant impact on animal health and welfare.

Currently, the most widely used test for detecting bTB is based on measurement of a delayed-type hypersensitivity (DTH) reaction to bovine tuberculin (purified protein derivative of *M. bovis*; PPDb) at the site of administration in the skin (i.e., tuberculin skin test, TST) [6]. An alternative method to enhance skin test specificity is based on simultaneous intradermal injections of PPDb and *M. avium* tuberculin (PPDa). This method, in which specific DTH responses are compared, is known as the single intradermal comparative cervical tuberculin test (SICCT). However, skin induration measurements must be conducted 72 h after administration [7], which requires a substantial investment of time and farmer compliance. Furthermore, the sensitivity and specificity of the test can be influenced by factors like operator bias and -competency, BCG vaccination, and co-infections with environmental nontuberculous mycobacterium (NTM) or parasites [8,9]. Tests relying on PPDb cannot differentiate between infected and vaccinated animals (DIVA), which limits the use of BCG in cattle [10]. Although the BCG vaccine has been used in humans for nearly a century and has shown effective protection against TB in animals through numerous experimental and field studies [11].

On the other hand, the interferon-gamma release assay (IGRA) is based on specific activation of Th1 cells in response to *M. bovis* antigens. The antigens used in commercially available IGRAs for bTB are PPDs [12], facing similar issues to those of the TST that hamper accuracy. Trials have been conducted using antigens present in *M. bovis* but absent in BCG, aiming to enhance specificity and offer DIVA capabilities [13]. However, the IGRA requires overnight incubation, trained lab personnel, as well as benchtop equipment [14], thus it cannot be used as a rapid test. Besides diagnostic tests based on cellular responses, several commercial immunoassays based on detection of antibodies are available, including the IDEXX *M. bovis* antibody (Ab) test, which targets MPB70 and MPB83 antigens. However, the sensitivity and specificity of these tests vary [15]. Therefore, the diagnosis of bTB remains challenging even in high income countries and the development of accurate, rapid and easy-to-use tests is warranted.

Phenolic glycolipids (PGLs) are located in the outermost layers of the mycobacterial envelope of major pathogenic mycobacteria [16]. Although PGLs share a common structure, species-specific differences are present [17,18]. The *M. tuberculosis* specific PGL, known as PGL-tb1, is produced only by certain hypervirulent strains (e.g. Beijing and Canetti stains) [19,20]. In a study conducted in Korea, 32 out of 50 clinical isolates contained this glycolipid [21]. In a Japanese study, specific antibodies against PGL-tb1 and its analogue were found in 28.8 % of active TB patients (*n* = 111), 4.0 % of older employees (average age 42.8, *n* = 50), 61.9 % of younger employees (average age 21.9, *n* = 42) undergoing health checkups, and 18.0 % of primary school students (*n* = 100) [22]. These findings indicate that anti-PGL-tb1 antibodies cannot be used to sensitively and specifically detect human TB. *M. leprae* PGL-I contains a trisaccharide that is highly specific for this mycobacterium as well as *M. lepromatosis* [23]. It has been shown in various studies that the detection of anti-*M. leprae* PGL-I antibodies in serum and fingerstick blood can accurately and specifically identify past or present *M. leprae* infection in humans [[24], [25], [26]]. Moreover, detecting anti-*M. leprae* PGL-I antibodies has also proven to be effective for detection of *M. leprae* in armadillos [27] and red squirrels [28].

**Fig. 1:**
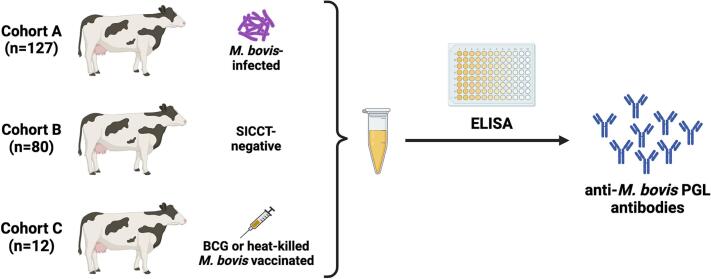
Study workflow. Levels of anti-*M. bovis* PGL antibody in serum samples from naturally infected cattle (Cohort A), SICCT-negative cattle (Cohort B), and cattle vaccinated with BCG or heat-killed *M. bovis* (Cohort C) were measured using ELISA.

Given the robust performance of *M. leprae* PGL-I serology in specifically detecting *M. leprae* infection in humans and animals, we aimed in this study to evaluate the potential of anti-*M. bovis* PGL antibodies as biomarkers for *M. bovis* infection in cattle by accessing seroprevalence in serum from different bovine cohorts (Fig. 1).

## 2 Materials and methods

### 2.1 Sample collection

*M. bovis*-infected cattle (Cohort A): serum samples (*n* = 127) were obtained from naturally *M. bovis*-infected cattle in the UK (Table S1). The SICCT was applied to all animals as the initial test to confirm infection, and sera from skin reactors were collected within 10–30 days post skin test. All animals had gross tuberculous lesions and were positive in both the SICCT test and *M. bovis* culture of lymph nodes or lung biopsies. All serum samples were tested using the IDEXX *M. bovis* Ab test as per the manufacturer's instructions upon sample collection.

SICCT-negative cattle (Cohort B): serum samples (*n* = 80) were obtained from a herd in the UK certified as officially TB-free with no TB positive cattle identified by the SICCT for >10 years (Table S2). All animals were confirmed SICCT negative, and blood was sampled approximately 30 days following the last SICCT.

Vaccinated cattle (Cohort C): serum samples from 12 vaccinated (BCG-vaccinated (*n* = 6) and heat-killed *M. bovis*-vaccinated (*n* = 6)) animals sampled as described previously [29] were used. BCG vaccinated animals received 2 × 10^6^ CFU live *M. bovis* BCG Danish 1331 (Statens Serum Institute, Denmark) at t_0_. Heat-killed *M. bovis* vaccinates received 1 × 10^7^ CFU heat-killed *M. bovis* at t_0_ and t_3_ [29].

Serum samples from leprosy patients with high anti-*M. leprae* PGL-I IgM levels (*n* = 20) were recruited at the Dept. Dermatology, Erasmus Medical Center, Rotterdam, The Netherlands [30].

### 2.2 Synthetic *M. bovis* PGL conjugates

The monosaccharide (2-*O*-methyl-α-L-rhamnopyranoside) [31] of *M. bovis* specific PGL (Fig. S1) were synthesized and coupled to bovine serum albumin (BSA) as described previously [[32], [33], [34]].

### 2.3 Production of recombinant protein MPT83

The *M. tuberculosis* gene MPT83 (Rv2873) exhibits 100 % query cover and identity of the sequence with MPB83 gene by BLASTN analysis [35]. The MPT83 gene was amplified by PCR from genomic DNA of *M. tuberculosis* and cloned using the Gateway technology platform (Invitrogen, Carlsbad, CA) with a pDEST17 expression vector containing an N-terminal histidine tag (Invitrogen). Sequencing was performed on selected clones to confirm the identities of all cloned DNA fragments. Recombinant protein MPT83 was overexpressed in *Escherichia coli* BL21 (DE3) and purified as described previously [36,37].

### 2.4 Detection of serum antibodies against MPT83 and *M. bovis* PGL by ELISAs

Bovine IgM and IgG levels specific for MPT83 or PGLs of *M. bovis* and *M. leprae* were measured in bovine sera by ELISA. Microlon plates (96 well, M2936, Sigma-Aldrich, St. Louis, MO) were coated with 100 μl of the MPT83 recombinant protein (5 μg/ml) or *M. bovis* PGL (2 μg/ml) in PBS and incubated at 37 °C for 2 h. Blocking solution (200 μl of PBS/1 % BSA/1 % Tween-20) was added to each well and incubated at 37 °C for 1 h. Serum samples (100 μl of 1:400 dilution) in PBS/1 % BSA/0.05 % Tween-20 were added and incubated at 4 °C for 16 h. Subsequently, 100 μl of rabbit anti-bovine IgG-HRP (1:8000, SAB3700020, Sigma-Aldrich) or mouse anti-bovine IgM-HRP (1:8000, clone: IL-A30, MCA2443PA, Bio-Rad, Hercules, CA) in PBS/1 % BSA/0.05 % Tween-20 was incubated at 37 °C for 2 h. The plates were washed three times (200 μl PBS/0.05 % Tween-20) between each step. Finally, 100 μl of 3,3′,5,5′-Tetramethylbenzidine (Thermo Fisher Scientific, Rochester, NY) was added. The colour reaction was stopped after 15 min using 100 μl H_2_SO_4_ (1 M). The optical density at 450 nm (OD_450_) of the samples was corrected for the background OD (0.4 % BSA in coating buffer).

### 2.5 Anti-*M. leprae* PGL-I IgM UCP-LFA

Anti-*M. leprae* PGL-I IgM UCP-LFA strips were prepared, and the assays were performed as described previously [38,39]. In brief, diluted serum samples (50 μl of a 1:50 dilution in assay buffer) were applied to lateral flow (LF) strips. Upon completion of LF, strips were analyzed with a UCP-dedicated benchtop reader (UPCON; Labrox, Finland). Results are displayed as the ratio value (R) of T and FC signals (peak area) based on relative fluorescence units (RFUs).

### 2.6 Statistical analyses

Graphpad Prism version 9.0.2 for Windows (GraphPad Software, CA) was used to perform Mann-Whitney *U* tests, Kruskal-Wallis with Dunn's correction for multiple testing, Fisher's exact test, plot receiver operating characteristic (ROC) curves, calculate the area under curve (AUC), and compute Spearman correlation coefficients. The statistical significance level used was *p* ≤ 0.05. The optimal sensitivity and specificity were determined using the Youden's index [40]. Venn diagrams were created using EVenn [41].

## 3 Results

### 3.1 Detection of anti-MPT83 antibody levels in cattle using ELISA

To determine dilution-conditions for sera and detecting reagents for assessment of bovine antibodies by ELISA, we first compared IgG responses against the in-house produced *Mtb* antigen, MPT83 (Rv2873), to IgG responses previously obtained for these sera in a commercially available bTB diagnostic test based on MPB83, which has 100 % identity with MPT83. Based on a dilution series of sera from *M. bovis*-infected (*n* = 24) and SICCT-negative animals (*n* = 24), the optimal serum- and detecting antibody (anti-bovine IgG) conditions were 1:400 and 1:8000, respectively, as significant levels of anti-MPT83 IgG were observed in the most of infected animals when using these dilutions, while OD_450_ for background (0.4 % BSA in PBS) remained below 0.1 (Fig. S2). Thus, these conditions were used in all subsequent experiments.

Serum samples from naturally *M. bovis*-infected cattle (Cohort A, *n* = 127) and SICCT-negative cattle (Cohort B, *n* = 80) in the UK were screened for anti-MPT83 IgG antibodies by ELISA. A cut-off value determined by Youden's index resulted in 74.8 % (95/127) seropositive animals among the *M. bovis*-infected cattle. This seropositivity rate showed no significant difference compared to using the IDEXX *M. bovis* Ab test (83.5 %, 106/127, cutoff value set as per manufacturer's instruction, Fig. S3, Table 1). Furthermore, 69.3 % (88/127) of the animals were positive in both tests (Fig. S3). Whereas 8.8 % (7/80) of animals tested seropositive for anti-MPT83 IgG (false-positive rate) in SICCT-negative cattle (Fig. S3, Table 1).

**Table 1:** Assay performance to discriminate *M. bovis*-infected and SICCT-negative cattle.

Method	Cut-off	Seropositivity vs culture positivity (true-positive rate)	Seropositivity vs SICCT-negative (false-positive rate)	ROC-AUC	*p* value
IDEXX *M. bovis* Ab test	S/P ratio ≥ 0.3	83.5 % (106/127)	N/A	N/A	N/A
anti-MPT83 IgG ELISA	OD_450-background_ > 0.185	74.8 % (95/127)	8.8 % (7/80)	0.85	<0.001
anti-*M. bovis* PGL IgG ELISA	OD_450-background_ > 0.705	48.8 % (62/127)	11.3 % (9/80)	0.70	<0.001
anti-*M. bovis* PGL IgM ELISA	OD_450-background_ > 0.395	44.9 % (57/127)	6.3 % (5/80)	0.71	<0.001
*M. bovis* PGL IgM + IgM MPT83 IgG	either test is positive	82.7 % (105/127)	15.0 % (12/80)	0.87	<0.001
*M. bovis* PGL IgM + IDEXX *M. bovis* Ab test	either test is positive	87.4 % (111/127)	N/A	N/A	N/A

**Fig. 2:**
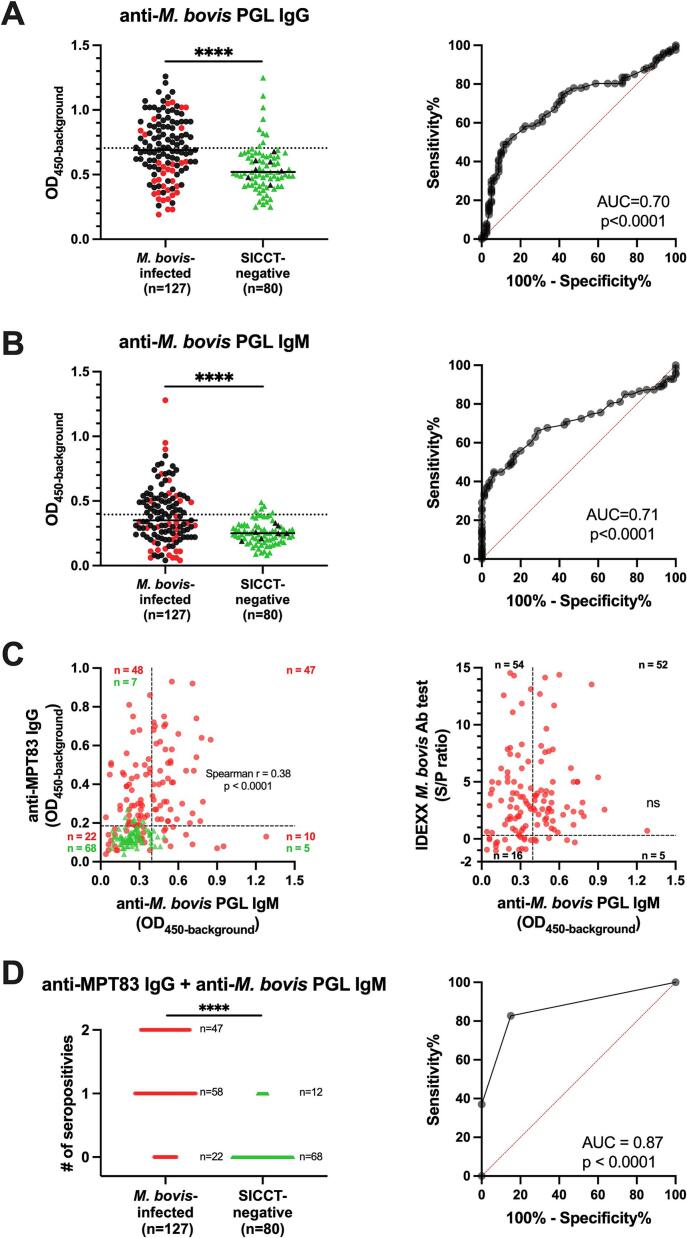
Differences in anti-*M. bovis* PGL antibody levels in serum samples from *M. bovis*-infected and SICCT-negative cattle. The anti-*M. bovis* PGL IgG (A) and -IgM (B) levels were measured by ELISA in sera from *M. bovis*-infected (*n* = 127, black/red dots indicate positive/negative in MPT83 IgG) and SICCT-negative cattle (*n* = 80, green triangles). ELISA results are displayed as optical density at 450 nm corrected for background OD values (OD_450-background_; *y-axis*). The median values of each group are indicated by horizontal lines. The results of anti-*M. bovis* PGL IgM vs. anti-MPT83 IgG ELISA for all cattle (described in Fig. S3) and vs. IDEXX *M. bovis* Ab test for *M. bovis*-infected cattle are shown in C. For the IDEXX test, sample-to-positive ratios (S/P) derived by subtracting the mean kit negative-control OD from each sample and dividing this value by the corrected positive-control value (mean positive-control OD minus mean negative-control OD). The S/P ≥ 0.3 (dashed line) is set as cut-off value according to the kit instruction. The index represents the number of positive results for these two markers (D). Difference between antibody levels in sera were determined by Mann-Whitney *U* tests; *P*-values: ∗∗∗∗*p* ≤ 0.0001. The ability to distinguish *M. bovis* infection in cattle was evaluated by ROC curve analysis, including AUC measurements (ROC-AUC) are shown in right panel. The cut-off value for anti-*M. bovis* PGL antibodies were determined by Youden's index and indicated by a dash line. (For interpretation of the references to colour in this figure legend, the reader is referred to the web version of this article.)

The sera from *M. bovis*-infected (*n* = 127) and SICCT-negative (*n* = 80) cattle were tested by antibody ELISAs (described in Fig. 2, S3). Additionally, the IDEXX *M. bovis* Ab test was used to assess antibody levels in *M. bovis*-infected cattle. The ability to distinguish different *M. bovis* infection states was evaluated by ROC curve analysis, including AUC measurements (ROC-AUC). The cut-off value for each test is described in Fig. 2, S3. S/P, sample-to-positive ratios; ROC, receiver operating characteristic curves; AUC, area under curve; N/A: (data) not applicable.

### 3.2 Detection of anti-*M. bovis* PGL antibody levels in cattle using ELISA

After determining whether similar ELISA conditions were optimal for detecting anti-*M. bovis* PGL IgM in cattle (Fig. S4), sera from the *M. bovis*-infected and SICCT-negative cattle were assessed for IgM and IgG levels against *M. bovis* PGL (Fig. 2). Although anti-*M. bovis* PGL antibodies were detected in both *M. bovis*-infected and SICCT-negative cattle, antibody levels in *M. bovis*-infected cattle were significantly higher (AUC: 0.71 and 0.70, *p* < 0.0001) (Fig. 2A, B, and Table 1). Applying cut-off values determined by Youden's index for this cohort, anti-*M. bovis* PGL IgM and -IgG seropositivity levels in infected animals were 44.9 % (57/127) and 48.8 % (62/127) in *M. bovis*-infected cattle, respectively. These seroprevalence data are lower than those obtained with anti-MPT83 IgG ELISA (74.8 %, 95/127) and the IDEXX *M. bovis* Ab test (83.5 %, 106/127). In contrast, the positive proportions of anti-*M. bovis* PGL IgM and -IgG detected in SICCT-negative animals were 6.3 % (5/80) and 11.3 % (9/80), respectively (Fig. 2A, B, S5, and Table 1). Since the anti-*M. bovis* PGL IgG has a higher false-positive rate than -IgM, and the highest level in SICCT-negative cattle is close to the highest value in *M. bovis*-infected cattle, we next focus only on evaluating the performance of anti-*M. bovis* PGL IgM.

The finding that the correlation between anti-*M. bovis* PGL IgM levels and anti-MPT83 IgG (*r* = 0.38, *p* < 0.001) was weak, indicates added value in using these tests in a combined fashion for diagnosis of *M. bovis* infection (Fig. 2C, S6, Table 1):

Combining the seropositivity data of both anti-*M. bovis* PGL IgM and anti-MPT83 IgG, in which a positive result is indicated by either of the tests or both being positive, the AUC increased from 0.71 of anti-*M. bovis* IgM to 0.87 (*p* < 0.0001) (Fig. 2D, Table 1).

**Fig. 3:**
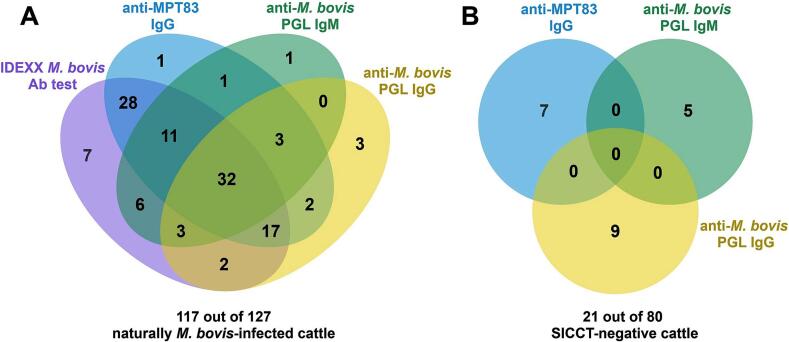
Correlation between seropositivity in different tests. Venn diagram depicting seropositivity in different tests in *M. bovis*-infected (*n* = 127) and SICCT-negative cattle (*n* = 80). 117 out of 127 *M. bovis*-infected cattle (A) and 14 out of 80 SICCT-negative cattle (B), respectively, each had at least one positive test result. The cut-off values for determinng seropositivity of each test are described in Fig. 2, S3. Each circle represents the presence of a positive result on a specific test. The overlap between the circles illustrates cases where two or more tests are positive concurrently. In *M. bovis*-infected cattle, 10 samples were negative in all tests, while SICCT-negative cattle had 59 samples test negative in all tests.

In *M. bovis*-infected cattle, using anti-MPT83 IgG or the IDEXX *M. bovis* Ab test combined with anti-*M. bovis* PGL IgM, the positive proportions increased from 74.8 % and 83.5 % to 82.7 % (105/127) (*p* < 0.001) and 87.4 % (111/127) (non-significant), respectively, with an additional 10 and five infected cattle identified, respectively (Fig. 2, Fig. 3, Table 1). Specificity of combining the two test results was slightly, but not significantly, lower (Fig. 2, Fig. 3, S5, and Table 1).

### 3.3 Anti-*M. bovis* PGL antibody levels in vaccinated cattle

**Fig. 4:**
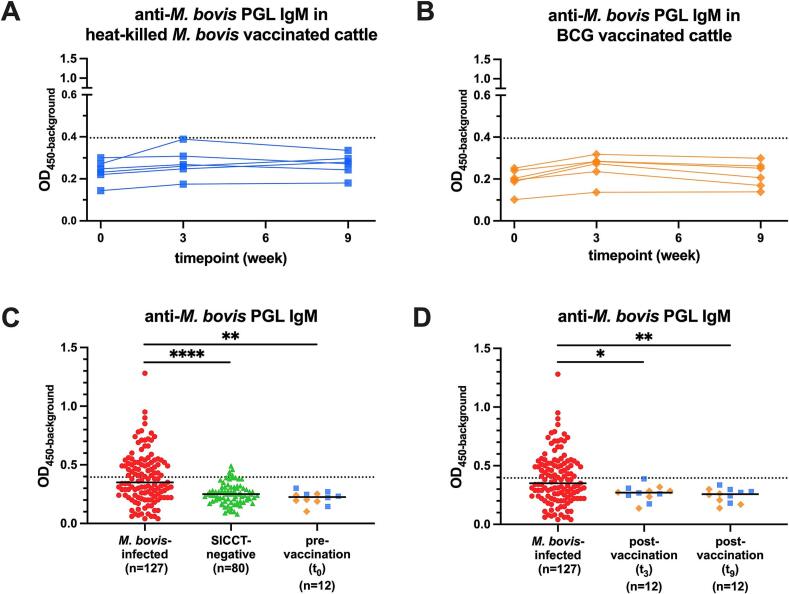
Anti-*M. bovis* PGL IgM levels in vaccinated cattle. The levels of anti-*M. bovis* PGL IgM in heat-killed *M. bovis*- (blue squares, *n* = 6, panel A) and BCG- (yellow rhombus, *n* = 6, panel B) vaccinated cattle were measured by ELISA. ELISA results are displayed as optical density at 450 nm corrected for background OD values (OD_450-background_; *y-axis*). Samples from the same animal are connected using polylines. Difference between time points were determined by Friedman test. BCG vaccinates had received BCG at week 0 (t_0_), whereas heat-killed *M. bovis* vaccinates received heat-killed *M. bovis* at week 0 (t_0_) and week 3 (t_3_). Sera were collected 0 weeks (t_0_) before, and 3 (t_3_), 9 (t_9_) weeks after prime vaccination. The cut-off value (described in Fig. 2) is indicated by a dash line. Serum levels of cattle pre-vaccination (t_0_) and post-vaccination (t_3_ and t_9_) compared to the other cohorts are shown in C and D panel, respectively. Difference between cohorts determined by Kruskal-Wallis with Dunn's correction for multiple testing, P-values: ∗*p* ≤ 0.05, ∗∗*p* ≤ 0.01, ∗∗∗∗*p* ≤ 0.0001. (For interpretation of the references to colour in this figure legend, the reader is referred to the web version of this article.)

To elucidate the impact of vaccination on anti-*M. bovis* PGL IgM levels and to assess the potential of this assay to differentiate infected from vaccinated animals (DIVA), we also examined the anti-*M. bovis* PGL antibody levels using ELISA in serum of South African cattle that were vaccinated with BCG or heat-killed *M. bovis* in a previous study (Cohort C). No significant changes in IgM levels were observed between baseline (t_0_), three weeks (t_3_), and nine weeks (t_9_) post-BCG or heat-killed *M. bovis* vaccination, although very low levels above background could be detected for anti-PGL IgM. (Fig. 4A, B). The serum antibody levels of cattle pre-vaccination (*p* < 0.01), or three (*p* < 0.05) and nine (*p* < 0.01) weeks post-vaccination were lower compared to those of *M. bovis*-infected cattle, and did not differ from the SICCT-negative cattle (Fig. 4C, D).

### 3.4 Cross-reaction between PGLs of different mycobacteria

**Fig. 5:**
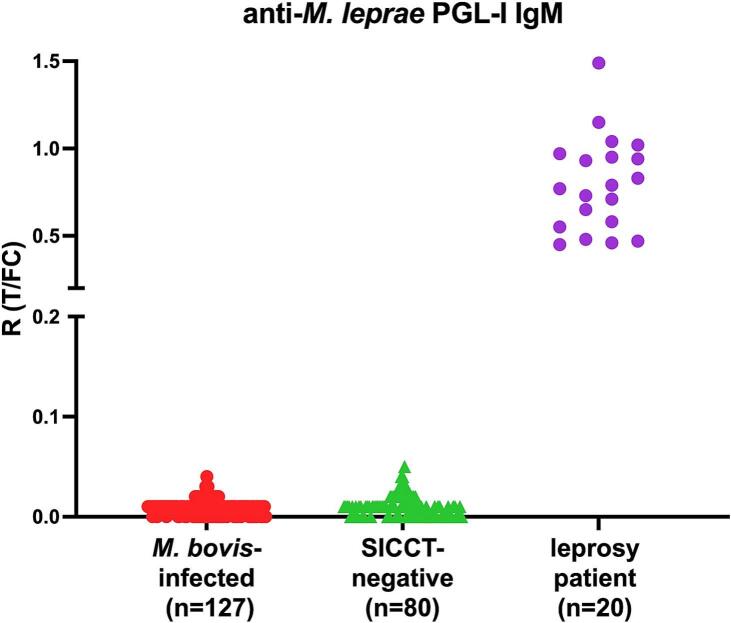
Detection of anti-*M. leprae* PGL-I IgM levels in bovine sera using UCP-LFA for human antibody detection. The anti-*M. leprae* PGL-I IgM levels were measured by UCP-LFA in serum from *M. bovis*-infected (red dots, *n* = 127) and SICCT-negative cattle (green triangles, *n* = 80), leprosy patients (purple dots, *n* = 20). The serum samples were diluted 1:50. UCP-LFA results are displayed as the Ratio value (R) between Test (T) and Flow-Control (FC) signal (*y-axis*). The sample with R ≥ 0.1 was considered positive [25]. (For interpretation of the references to colour in this figure legend, the reader is referred to the web version of this article.)

To assess potential cross-reactivity for PGLs corresponding to different mycobacteria, we also tested bovine serum samples using the rapid quantitative (QR)-test for detection of anti-*M. leprae* PGL-I IgM based on up-converting reporter particle lateral flow assays (UCP-LFAs) [38,39,42,43]. None of the bovine serum samples tested positive (Fig. 5). This indicates that no cross-reactivity for *M. leprae* PGL-I was detected in *M. bovis* infected cattle.

## 4 Discussion

Development of accurate diagnostic tests for *M. bovis* infection is essential to address the significant One Health issues caused by infection with this pathogen. Infection of cattle with *M. bovis* causes large economic losses from bTB and poses a significant threat to human health due to zoonotic transmission, especially in LMICs [3,5]. Bacterial culture remains the gold-standard for bTB, but it is relatively expensive, time-consuming (taking several weeks), and requires a biosafety level 3 laboratory [44], making it impractical for LMICs.

Drawing inspiration from the success of anti-*M. leprae* PGL-I antibodies for detecting *M. leprae* infection in humans [[24], [25], [26]] and animals [27,28], our study investigated the potential of anti-*M. bovis* PGL antibodies for detecting *M. bovis* infection.

Anti-*M. bovis* PGL antibodies were specifically detected in serum of *M. bovis*-infected cattle although sensitivity was low. However, when setting the cutoff to ensure 100 % specificity, i.e. eliminating all false positives in a screening in TB-free herd, the sensitivity drops significantly from 44.9 % (57/127) to 29.13 % (37/127) (*p* < 0.05) which highlights a critical trade-off between achieving absolute specificity and retaining sufficient sensitivity for disease detection. This is crucial for bTB detection, especially in the context of test-and-slaughter policy, where an excessively high false-positivity rate could lead to unnecessary economic losses.

Using either anti-*M. bovis* PGL IgM or anti-MPT83 IgG individually only provided moderate sensitivity for detecting infection. The detection of antibodies to *M. bovis* PGL IgM falls short of achieving the same high level of reliability observed with *M. leprae* PGL-I in diagnosing infection in humans, indicating a divergence in the host immune response to PGLs between *M. bovis* and *M. leprae* infection.

Despite observing a reduction in seropositivity rates in infected cattle when assessed individually, the combined detection of both anti-*M. bovis* PGL IgM and anti-MPT83 IgG (not significantly) increased the seropositivity rate in infected cattle while maintaining specificity. This underscores the notion that the anti-*M. bovis* PGL IgM, detects in part different animals, and therefore holds promise in a multi-antigen approach and may be pivotal in further enhancing the method's efficiency.

Development of new diagnostic tests with reliable DIVA capability is crucial for advancing vaccine research and application. The IDEXX *M. bovis* Ab test, while capable of differentiating *M. bovis* infection from BCG vaccination, still faces challenges in distinguishing animals vaccinated with heat-killed *M. bovis*, a promising newly developed vaccine candidate [29,45], from infected animals. Our results show that the serum levels of anti-*M. bovis* PGL IgM and its reliability as a biomarker for *M. bovis* infection were unaffected by both live BCG and heat-killed *M. bovis* vaccination.

However, due to the inherent limitations in the accuracy of SICCT itself, there may be potential risks in the specificity analysis of the SICCT-negative cattle. In line with these findings, low levels of anti-*M. bovis* PGL IgM and -IgG were also detected in some of the SICCT-negative cattle, albeit in different cattle (Fig. 3). Since other mycobacteria such as *M. microti, M. pinnipedii, M. africanum*, *M. ulcerans* also contain a highly similar structure of PGL as *M. bovis* [46,47], anti-*M. bovis* PGL seropositivity may be attributed to exposure to other mycobacteria in the natural environment. On the other hand, some NTM, such as *M. kansasii*, contain a different PGL structure (PGL-K1) [48], which may help address the issue of cross-reactivity with MPB83-based tests [49], This underscores the importance of combining multiple mycobacterial target antigens in diagnostic tools. Testing samples from cattle infected with different mycobacterial species will be necessary to gain more insight into the role of PGL-specific antibodies in animals and utilize their diagnostic value more efficiently.

However, using *M. leprae* PGL-I as a target antigen, as developed for detection of *M. leprae* infection in humans, *M. bovis*-infected cattle did not test positive for anti-*M. leprae* PGL-I by UCP-LFA. This confirms the specificity of the diagnostic tests for *M. leprae* infection [25,38,39,42,43].

### 4.1 Limitation of the study

In view of the low sensitivity for *M. bovis* infection of anti-*M. bovis* PGL antibodies, a limitation of this study was the limited number available of serum samples available from infected cattle of the same breed, as well as the lack of samples from cattle infected with other mycobacteria. In addition, the lack of pathology scores of infected animals impeded the determination of quantitative associations between anti-*M. bovis* PGL antibody levels and lung pathology. Also, further research is necessary to address the potential influence of other mycobacteria in the environment and determine the combined diagnostic potential of anti-PGL antibodies with other host biomarkers such as cytokines [50].

## 5 Conclusion

This study is the first to detect antibodies directed against *M. bovis* PGL in *M. bovis* infected cattle, and demonstrates that these antibodies, exhibit high specificity for *M. bovis* infection in cattle. However, their sensitivity is moderate and therefore has much less diagnostic potential than anti-*M. leprae* PGL-I IgM displays for multibacillary leprosy. Therefore, while anti-*M. bovis* PGL IgM can serve as a potential biomarker for *M. bovis* infection, it should be used in combination with other markers to further enhance diagnostic performance.

## Ethics statement

The cattle in Cohort A and C were approved by the APHA Animal Welfare and Ethical Review Board (reference PF7D840A5–3-001v2), and Animal Use and Care Committee of the University of Pretoria (Certificate number V066–15), respectively. Cattle in Cohort B were sampled as part of an ongoing clinical diagnostic study of Johne's Disease within the herd. Blood sampling was carried out by the Millcroft Veterinary Group Ltd., Cumbria, UK and remnant blood samples were provided for this study. The use of human leprosy sera was approved by medical ethics committee of the Erasmus Medical Center (MEC-2012-589).

## Funding

This study was supported with grants from the Q.M. Gastmann-Wichers Foundation (to AG), Zijie Zhou was supported with grant from the China Scholarship Council. JCH was funded by Biotechnology and Biological Sciences Research Council Institute Strategic Programme funding (BBS/E/RL/230002B). HK was funded by a joint PhD studentship from the University of Edinburgh, United Kingdom, and Leiden University Medical Center, The Netherlands.

## CRediT authorship contribution statement

**Zijie Zhou:** Writing – review & editing, Writing – original draft, Investigation, Formal analysis, Data curation. **Anouk van Hooij:** Writing – review & editing, Formal analysis. **J. Hessel M. van Dijk:** Writing – review & editing, Resources. **Nina Musch:** Writing – review & editing, Resources. **Louise Pierneef:** Writing – review & editing, Resources. **Hamza Khalid:** Writing – review & editing, Investigation, Data curation. **Kees Franken:** Writing – review & editing, Resources. **Thomas Holder:** Writing – review & editing, Investigation, Data curation. **Neil Watt:** Writing – review & editing, Resources. **Anita L. Michel:** Writing – review & editing, Resources. **Jeroen D.C. Codée:** Writing – review & editing, Resources. **Martin Vordermeier:** Writing – review & editing. **Paul L.A.M. Corstjens:** Writing – review & editing, Resources. **Elisabeth M.D.L. van der Heijden:** Writing – review & editing, Data curation. **Jayne C. Hope:** Writing – review & editing, Data curation. **Annemieke Geluk:** Writing – review & editing, Writing – original draft, Formal analysis, Conceptualization.

## Declaration of competing interest

The authors declare that the research was conducted in the absence of any commercial or financial relationships that could be construed as a potential conflict of interest.

## Data Availability

The original contributions presented in the study are included in the article/supplementary material.

## Appendix A Supplementary data

Supplementary material Image 1
