# Supplementary material for: Anti-phenolic glycolipid antibodies in Mycobacterium bovis infected cattle
Source: One Health. 2025 Jan 28;20:100982. doi: 10.1016/j.onehlt.2025.100982 (PMC11835577; doi:10.1016/j.onehlt.2025.100982)
Supplement: Supplementary file 1 — Supplementary material [file mmc1.docx]

***Supplementary Materials***

**Anti- phenolic glycolipid antibodies**

**in *Mycobacterium bovis* infected cattle**

Zijie Zhou, Anouk van Hooij, J. Hessel M. van Dijk, Nina Musch,

Louise Pierneef, Hamza Khalid, Kees Franken, Thomas Holder, Neil Watt,

Anita L. Michel, Jeroen D.C. Codée, Martin Vordermeier,

Paul L.A.M. Corstjens, Elisabeth M.D.L. van der Heijden, Jayne C. Hope, and Annemieke Geluk

***Supplementary Table S1: Characteristics of M. bovis-infected cattle cohort***

| **Sample No.** | **Gender** | **Breed code** | **Age** | **Lesion location: Head (H), Lung (L), alimentary tract (A)** | **Laboratory culture of *M. bovis*** | **Typing information of *M. bovis*** | **SICCT** |
| --- | --- | --- | --- | --- | --- | --- | --- |
| 1 | Female | LIMX | 1 year | L | positive | Spoligo type9 | positive |
| 2 | Male | SAX | 1 year 7 months | H | positive | Spoligo type9 | positive |
| 3 | Male | LIMX | 2 years 2 months | H | positive | Spoligo type9 | positive |
| 4 | Male | LIMX | 2 years 3 months | H | positive | Spoligo type9 | positive |
| 5 | Male | LIMX | 2 years 3 months | H | positive | Spoligo type9 | positive |
| 6 | Male | LIMX | 2 years 2 months | H | positive | Spoligo type9 | positive |
| 7 | Female | BBX | 1 year 2 months | H | positive | Spoligo type9 | positive |
| 8 | Male | LIMX | 1 year | H | positive | Spoligo type9 | positive |
| 9 | Male | LIMX | 2 years 4 months | H | positive | Spoligo type9 | positive |
| 10 | Male | LIMX | 2 years | H | positive | Spoligo type9 | positive |
| 11 | Male | LIMX | 2 years 1 month | H | positive | Spoligo type9 | positive |
| 12 | Female | FR | 10 years 2 months | L | positive | Spoligo type17 | positive |
| 13 | Male | LIMX | 2 years 5 months | H | positive | Spoligo type9 | positive |
| 14 | Male | CHX | 2 years | H | positive | Spoligo type9 | positive |
| 15 | Male | LIMX | 1 year 7 months | H | positive | Spoligo type9 | positive |
| 16 | Male | CHX | 2 years 3 months | H | positive | Spoligo type9 | positive |
| 17 | Female | DEX | 1 years 3 months | A | positive | Spoligo type17 | positive |
| 18 | Male | WB | 2 years 3 months | H, L & A | positive | na | positive |
| 19 | Male | LIMX | 2 years 4 months | H | positive | Spoligo type9 | positive |
| 20 | Female | HF | 4 years 9 months | H, L & A | positive | Spoligo type9 | positive |
| 21 | Male | CHX | 2 years | H | positive | Spoligo type9 | positive |
| 22 | Male | LIMX | 2 years | H, L & A | positive | Spoligo type9 | positive |
| 23 | Male | LIMX | 2 years 1 month | H, L & A | positive | Spoligo type9 | positive |
| 24 | Male | HO | 3 years 1 month | H, L & A | positive | Spoligo type9 | positive |
| 25 | Male | LIMX | 2 years | H | positive | Spoligo type9 | positive |
| 26 | Male | HF | 1 year 4 months | H | positive | Spoligo type9 | positive |
| 27 | Male | CHX | 2 years 2 months | H, L & A | positive | Spoligo type9 | positive |
| 28 | Female | BH | 3 years | H, L & A | positive | Spoligo type9 | positive |
| 29 | Female | HF | 4 years 3 months | H, L & A | positive | Spoligo type9 | positive |
| 30 | Female | HF | 3 years | H | positive | Spoligo type9 | positive |
| 31 | Female | HO | 5 years | H, L & A | positive | na | positive |
| 32 | Female | HO | 9 years | H | positive | na | positive |
| 33 | Female | HO | 4 years | H | positive | Spoligo type9 | positive |
| 34 | Male | HF | 1 year | H, L & A | positive | Spoligo type17 | positive |
| 35 | Female | LIMX | 2 years | H, L & A | positive | Spoligo type9 | positive |
| 36 | Female | LIMX | 1 year 9 months | H, L & A | positive | Spoligo type9 | positive |
| 37 | Male | HF | 2 years | H | positive | Spoligo type9 | positive |
| 38 | Female | LIMX | 2 years | H, L & A | positive | Spoligo type9 | positive |
| 39 | Male | HF | 1 year | H, L & A | positive | Spoligo type17 | positive |
| 40 | Female | HX | 12 years 7 months | H, L & A | positive | Spoligo type9 | positive |
| 41 | Male | LIMX | 2 years | H, L & A | positive | na | positive |
| 42 | Female | HF | 4 years 7 months | L | positive | Spoligo type9 | positive |
| 43 | Female | SM | 1 year 4 months | L | positive | Spoligo type17 | positive |
| 44 | Female | HF | 2 years 9 months | L | positive | Spoligo type9 | positive |
| 45 | Female | HEX | 2 years | H | positive | Spoligo type9 | positive |
| 46 | Female | HEX | 1 year 3 months | H | positive | Spoligo type9 | positive |
| 47 | Female | HEX | 1 year 3 months | L | positive | na | positive |
| 48 | Female | HEX | 2 years 3 months | H & L | positive | Spoligo type9 | positive |
| 49 | Female | BBX | 1 year 7 months | L | positive | Spoligo type9 | positive |
| 50 | Female | BBX | 1 year 7 months | L | positive | Spoligo type9 | positive |
| 51 | na | na | na | na | positive | Spoligo type9 | positive |
| 52 | na | na | na | na | positive | Spoligo type9 | positive |
| 53 | Female | LIM | 1 year 4 months | H | positive | Spoligo type9 | positive |
| 54 | Male | WBX | 2 years 4 months | L | positive | Spoligo type9 | positive |
| 55 | na | na | 10 years 7 months | A | positive | Spoligo type9 | positive |
| 56 | Female | LIM | 1 year 9 months | H | positive | Spoligo type9 | positive |
| 57 | Female | SMX | 2 years 3 months | L | positive | Spoligo type9 | positive |
| 58 | na | na | 10 years 11 months | A | positive | Spoligo type9 | positive |
| 59 | Female | LIMX | 1 year 1 month | A | positive | Spoligo type17 | positive |
| 60 | na | na | 1 year 9 months | A | positive | Spoligo type9 | positive |
| 61 | Female | CHX | 1 year 11 months | L | positive | Spoligo type9 | positive |
| 62 | Male | LIMX | 2 years | L | positive | Spoligo type9 | positive |
| 63 | Female | HEX | 2 years | L | positive | Spoligo type9 | positive |
| 64 | Male | LIMX | 2 years | L | positive | Spoligo type9 | positive |
| 65 | Female | CHX | 2 years | L | positive | Spoligo type9 | positive |
| 66 | Male | CHX | 2 years | L | positive | Spoligo type9 | positive |
| 67 | Female | HEX | 1 year 10 months | A | positive | Spoligo type9 | positive |
| 68 | Female | CHX | 1 year 11 months | L | positive | Spoligo type9 | positive |
| 69 | Male | HEX | 2 years | L | positive | Spoligo type9 | positive |
| 70 | Male | LIMX | 2years | L | positive | Spoligo type9 | positive |
| 71 | Female | CHX | 2 years | A | positive | Spoligo type9 | positive |
| 72 | Male | CHX | 2 years | L | positive | Spoligo type9 | positive |
| 73 | Male | CHX | 10 months | A | positive | na | positive |
| 74 | Male | CHX | 1 year | A | positive | Spoligo type9 | positive |
| 75 | Male | SMX | 1 year 1 month | L | positive | na | positive |
| 76 | Female | CHX | 1 year | A | positive | na | positive |
| 77 | Female | CHX | 1 year | L & A | positive | Spoligo type9 | positive |
| 78 | Female | CHX | 2 years | L | positive | Spoligo type9 | positive |
| 79 | Male | CHX | 1 year | L | positive | Spoligo type9 | positive |
| 80 | Male | CHX | 1 year | L & A | positive | Spoligo type9 | positive |
| 81 | Male | CHX | 1 year | L | positive | Spoligo type9 | positive |
| 82 | Male | CHX | 2 years | L | positive | Spoligo type9 | positive |
| 83 | Female | CHX | 1 year | A & L | positive | Spoligo type9 | positive |
| 84 | Female | LIMX | 3 years | L | positive | Spoligo type9 | positive |
| 85 | Female | CHX | 2 years 11 months | A | positive | Spoligo type9 | positive |
| 86 | Male | CHX | 2 years 11 months | L | positive | Spoligo type9 | positive |
| 87 | Female | HEX | 3 years 2 months | A & L | positive | Spoligo type9 | positive |
| 88 | Female | HEX | 6 years 3 months | L | positive | Spoligo type9 | positive |
| 89 | Female | HEX | 5 years | L | positive | Spoligo type9 | positive |
| 90 | Female | HEX | 4 years 5 months | A | positive | Spoligo type9 | positive |
| 91 | Female | HEX | 10 years 5 months | L | positive | Spoligo type9 | positive |
| 92 | Female | HEX | 4 years 7 months | A | positive | Spoligo type9 | positive |
| 93 | Female | HEX | 11 years | L | positive | Spoligo type9 | positive |
| 94 | Female | HEX | 6 years | A | positive | Spoligo type9 | positive |
| 95 | Female | HEX | 6 years 6 months | L | positive | na | positive |
| 96 | Female | HF | 4 years 6 months | A | positive | Spoligo type9 | positive |
| 97 | Female | HF | 2 years 7 months | L | positive | Spoligo type9 | positive |
| 98 | Female | HF | 1 year 8 months | A | positive | Spoligo type9 | positive |
| 99 | Female | HF | 2 years 1 month | L&A | positive | Spoligo type9 | positive |
| 100 | Female | HF | 2 years 10 months | A | positive | Spoligo type9 | positive |
| 101 | Female | BF | 4 years 4 months | A | positive | Spoligo type11 | positive |
| 102 | Female | HF | 3 years | A | positive | Spoligo type9 | positive |
| 103 | Female | HF | 2 years 10 months | H | positive | Spoligo type9 | positive |
| 104 | Female | HF | 3 years | A | positive | Spoligo type9 | positive |
| 105 | Female | HF | 2 years 11 months | A | positive | Spoligo type9 | positive |
| 106 | Female | HE | 2 years 10 months | A | positive | Spoligo type9 | positive |
| 107 | Female | HE | 1 year 6 months | A | positive | Spoligo type9 | positive |
| 108 | Male | HEX | 1 year | H | positive | na | positive |
| 109 | Male | HEX | 1 year | H | positive | na | positive |
| 110 | Male | HEX | 1 year | H | positive | na | positive |
| 111 | Female | AAX | 2 years | H | positive | na | positive |
| 112 | Female | HFX | 2 years | H | positive | na | positive |
| 113 | Male | AAX | 2 years | H | positive | na | positive |
| 114 | Male | AAX | 2 years | H | positive | na | positive |
| 115 | Female | AAX | 2 years | H | positive | na | positive |
| 116 | Male | BRBX | 2 years | H | positive | na | positive |
| 117 | Female | HFX | 2 years | H | positive | na | positive |
| 118 | Female | HF | 2 years | H | positive | na | positive |
| 119 | Male | AAX | 1 year | H | positive | na | positive |
| 120 | Female | HF | 7 years | H | positive | na | positive |
| 121 | Female | BRBX | 7 months | H | positive | na | positive |
| 122 | Female | HF | 7 months | H | positive | na | positive |
| 123 | Female | HF | 1 year | H | positive | na | positive |
| 124 | Female | HF | 1 year | H | positive | na | positive |
| 125 | Male | BRBX | 5 months | L | positive | na | positive |
| 126 | Female | HEX | 1 year | H | positive | na | positive |
| 127 | Female | HEX | 1 year | H | positive | na | positive |

*Samples were obtained from 127 cattle (73 females; 49 males, 5 unknown) confirmed to be naturally M. bovis-infected using the single intradermal comparative cervical tuberculin test (SICCT) and the laboratorial culture. The cattle breeds and codes used in table are available at https://www.gov.uk/guidance/official-cattle-breeds-and-codes. Sera of 1-107 animals of this cohort were collected at an abattoir, and for each animal the laboratorial culture results of a lymph node pool were positive for M. bovis. Sera of 108-127 animals in this cohort, individual lymph nodes plus a lung biopsy were cultured, and a positive M. bovis culture result was obtained for each animal from at least one node. na, not applicable.*

***Supplementary Table S2: Characteristics of SICCT-negative cattle cohort***

| **Sample No.** | **Breed code** | **Gender** | **SICCT** |
| --- | --- | --- | --- |
| 1 | MOX | Female | Negative |
| 2 | BFX | Female | Negative |
| 3 | MO | Female | Negative |
| 4 | AYX | Female | Negative |
| 5 | BFX | Female | Negative |
| 6 | HFX | Female | Negative |
| 7 | AYX | Female | Negative |
| 8 | MOX | Female | Negative |
| 9 | AYX | Female | Negative |
| 10 | MOX | Female | Negative |
| 11 | AYX | Female | Negative |
| 12 | HFX | Female | Negative |
| 13 | SMX | Female | Negative |
| 14 | MOX | Female | Negative |
| 15 | PAX | Female | Negative |
| 16 | MOX | Female | Negative |
| 17 | HFX | Female | Negative |
| 18 | AYX | Female | Negative |
| 19 | AYX | Female | Negative |
| 20 | AYX | Female | Negative |
| 21 | AYX | Female | Negative |
| 22 | MOX | Female | Negative |
| 23 | HFX | Female | Negative |
| 24 | BFX | Female | Negative |
| 25 | MOX | Female | Negative |
| 26 | BFX | Female | Negative |
| 27 | BF | Female | Negative |
| 28 | BFX | Female | Negative |
| 29 | AYX | Female | Negative |
| 30 | MOX | Female | Negative |
| 31 | MOX | Female | Negative |
| 32 | AYX | Female | Negative |
| 33 | BFX | Female | Negative |
| 34 | HFX | Female | Negative |
| 35 | HFX | Female | Negative |
| 36 | HO | Female | Negative |
| 37 | MOX | Female | Negative |
| 38 | HFX | Female | Negative |
| 39 | AYX | Female | Negative |
| 40 | BFX | Female | Negative |
| 41 | MOX | Female | Negative |
| 42 | BFX | Female | Negative |
| 43 | MOX | Female | Negative |
| 44 | BFX | Female | Negative |
| 45 | AY | Female | Negative |
| 46 | JE | Female | Negative |
| 47 | BF | Female | Negative |
| 48 | AYX | Female | Negative |
| 49 | MO | Female | Negative |
| 50 | BF | Female | Negative |
| 51 | HFX | Female | Negative |
| 52 | MOX | Female | Negative |
| 53 | MOX | Female | Negative |
| 54 | BF | Female | Negative |
| 55 | AYX | Female | Negative |
| 56 | BFX | Female | Negative |
| 57 | BF | Female | Negative |
| 58 | BFX | Female | Negative |
| 59 | BF | Female | Negative |
| 60 | MOX | Female | Negative |
| 61 | BFX | Female | Negative |
| 62 | AYX | Female | Negative |
| 63 | BF | Female | Negative |
| 64 | BF | Female | Negative |
| 65 | BFX | Female | Negative |
| 66 | AYX | Female | Negative |
| 67 | BFX | Female | Negative |
| 68 | BFX | Female | Negative |
| 69 | AYX | Female | Negative |
| 70 | BFX | Female | Negative |
| 71 | BFX | Female | Negative |
| 72 | BFX | Female | Negative |
| 73 | HFX | Female | Negative |
| 74 | AYX | Female | Negative |
| 75 | BFX | Female | Negative |
| 76 | MOX | Female | Negative |
| 77 | MOX | Female | Negative |
| 78 | MOX | Female | Negative |
| 79 | SMX | Female | Negative |
| 80 | MOX | Female | Negative |

*Samples were obtained from 80 female cattle confirmed to be negative using the SICCT. The cattle breeds and codes used in table are available at https://www.gov.uk/guidance/official-cattle-breeds-and-codes.*

**
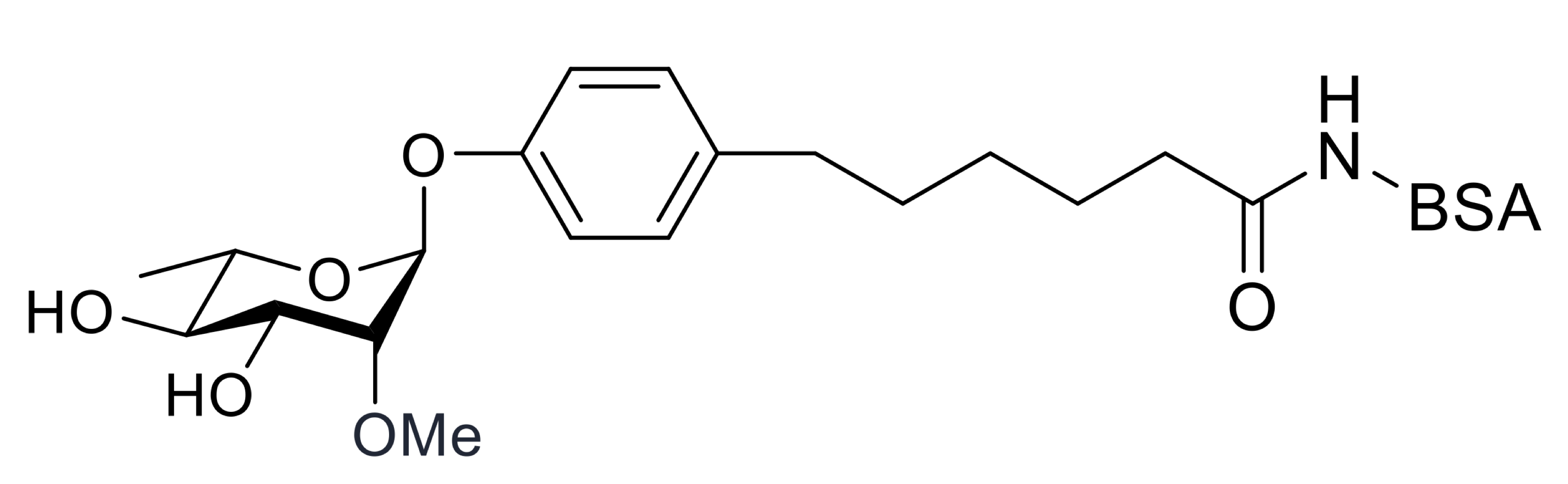
**

***Figure S1. The structure of synthetic M. bovis PGL monosaccharide****. Approximate average glycan loadings (based on MALDI analysis) were 39 monosaccharides per BSA molecule [1-3].*

**
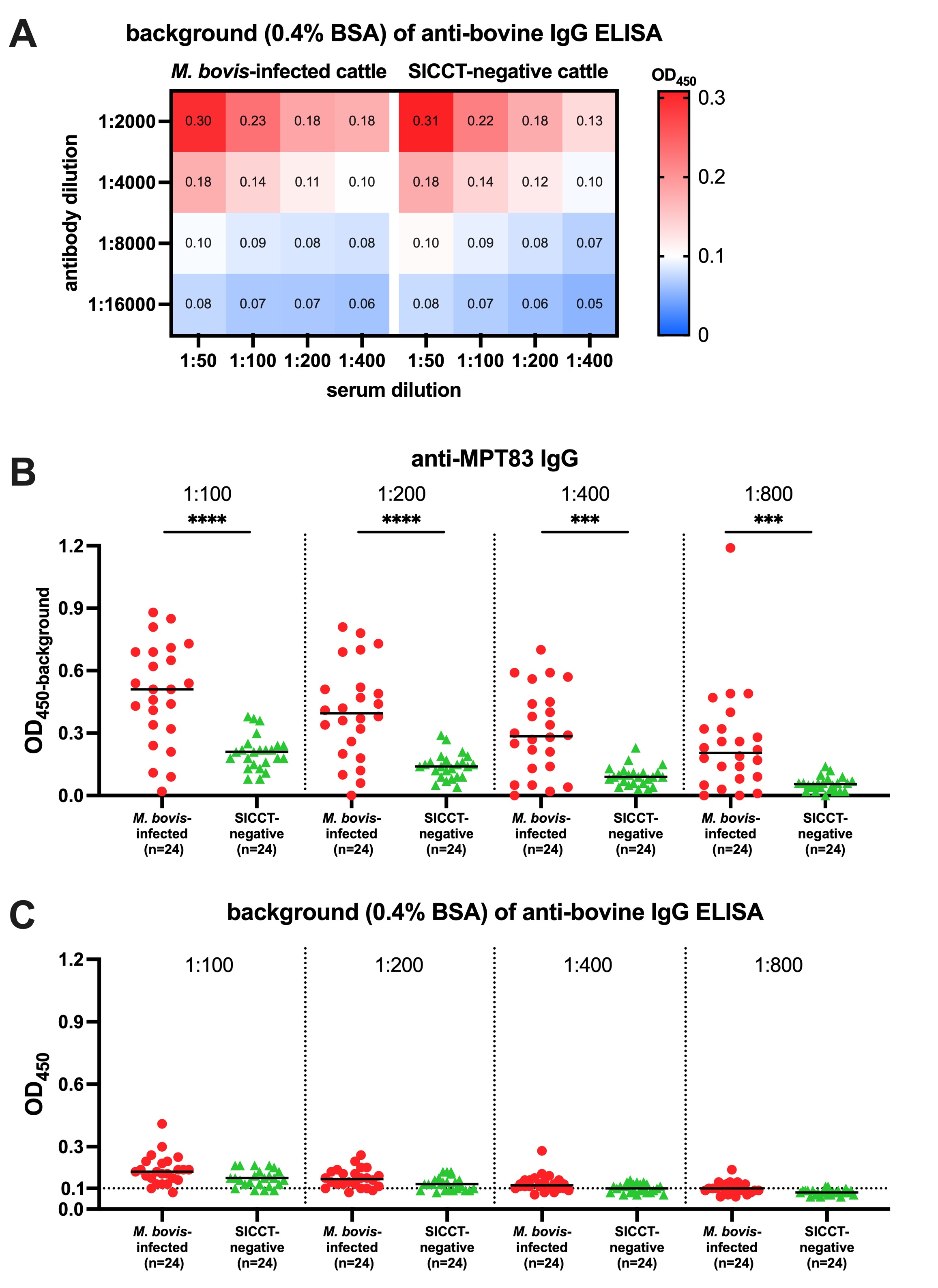
**

***Figure S2. Anti-bovine IgG background and anti-MPB83 IgG signals in ELISAs for different serum dilutions.*** *Heatmap depicting background (0.4% BSA coated) OD values of one random sample in M. bovis-infected and SICCT-negative cattle, respectively (#15 in Table S1 and #15 in Table S2), measured by ELISA at different detection antibody (1:2000 - 1:16000) and serum dilutions (1:50 - 1:400)* *(****A****). OD values for background and anti-MPB83 IgG (500 ng/well coated) in 24 randomly selected serum sample from M. bovis-infected (#24-47 in Table S1) and SICCT-negative (#21-44 in Table S2) cattle, respectively, were measured by ELISA of different serum dilutions (1:100 - 1:800). The anti-bovine IgG antibody was diluted 1:8000. ELISA results of background values are displayed as optical densities at 450 nm (OD_450;_ y-axis in* ***C****); anti-MPB83 IgG levels are displayed as optical density at 450 nm corrected for background OD values (OD_450-background_; y-axis in* ***B****). The median values of each group are indicated by horizontal lines; difference between antibody levels in sera were determined by Mann-Whitney U-tests; P-values: ∗∗∗p < 0.001, ∗∗∗∗p < 0.0001. inf., infected; neg., negative* *(****B****,* ***C****).*

**
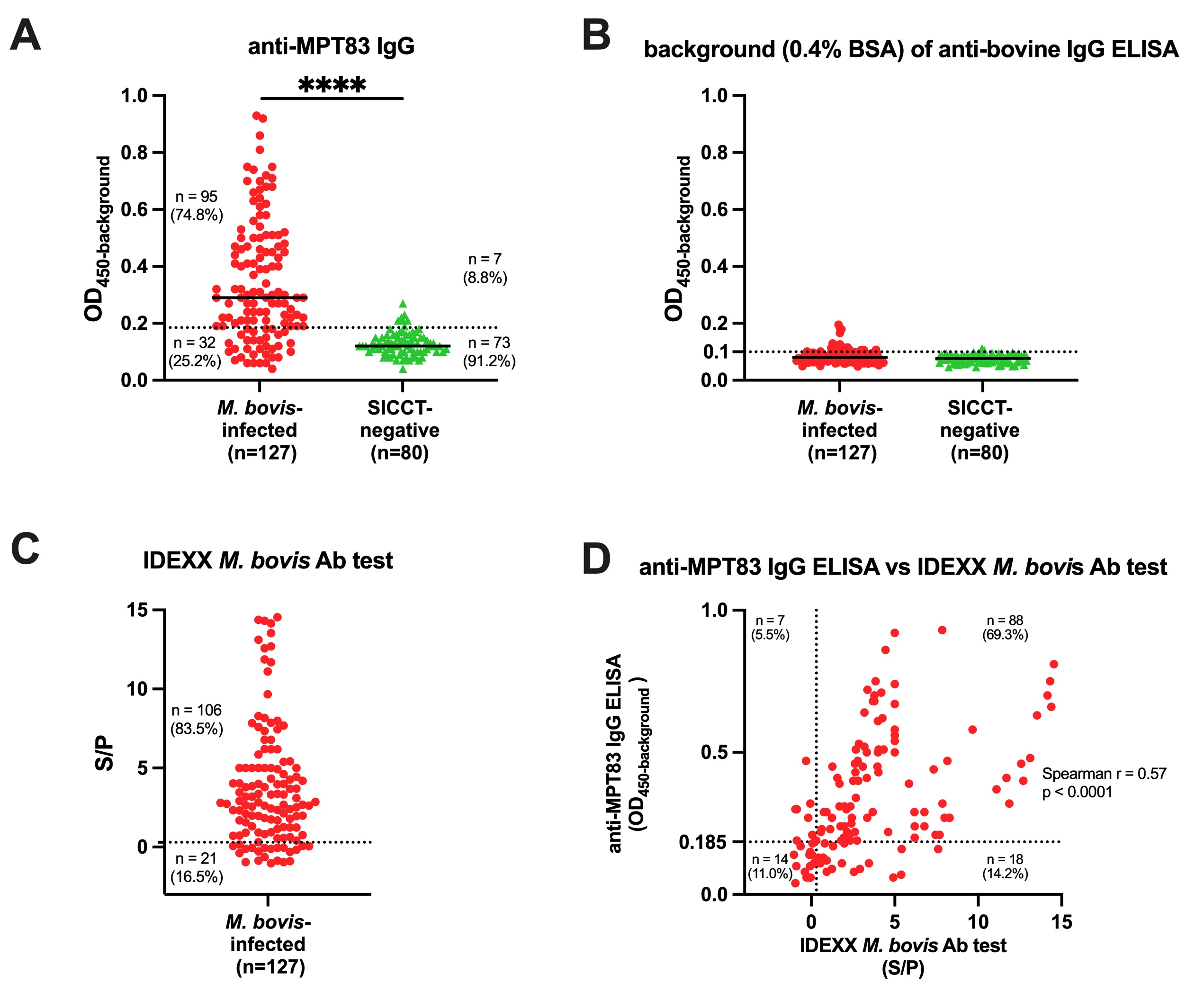
**

***Figure S3. Anti-MPT83 IgG levels in serum samples from naturally M. bovis-infected and SICCT-negative cattle.*** *Anti-MPT83 IgG (****A****) and background (0.4% BSA,* ***B****) levels were measured by ELISA in sera from M. bovis-infected (n=127, red dots) and SICCT-negative cattle (n=80, green triangles). ELISA results are displayed as optical density at 450 nm corrected for background OD values (OD_450-background_; y-axis). The cut-off value for anti-M. bovis PGL antibodies were determined by Youden’s index (OD_450-background_ >0.185, indicated by dashed line in* ***A****). Difference between antibody levels in sera were determined by Mann-Whitney U-tests; P-values: ∗∗∗∗p ≤ 0.0001. IDEXX M. bovis Ab test results (****C****) are presented as* *sample-to-positive ratios (S/P) derived by subtracting the mean kit negative-control optical density (OD) from each sample and dividing this value by the corrected positive-control value (mean positive-control OD minus mean negative-control OD). The S/P ≥ 0.3 (dashed line in* ***C****) is set as cut-off value according to the kit instruction. The correlation between anti-MPT83 IgG ELISA and IDEXX M. bovis Ab test is shown in panel* ***D****.*


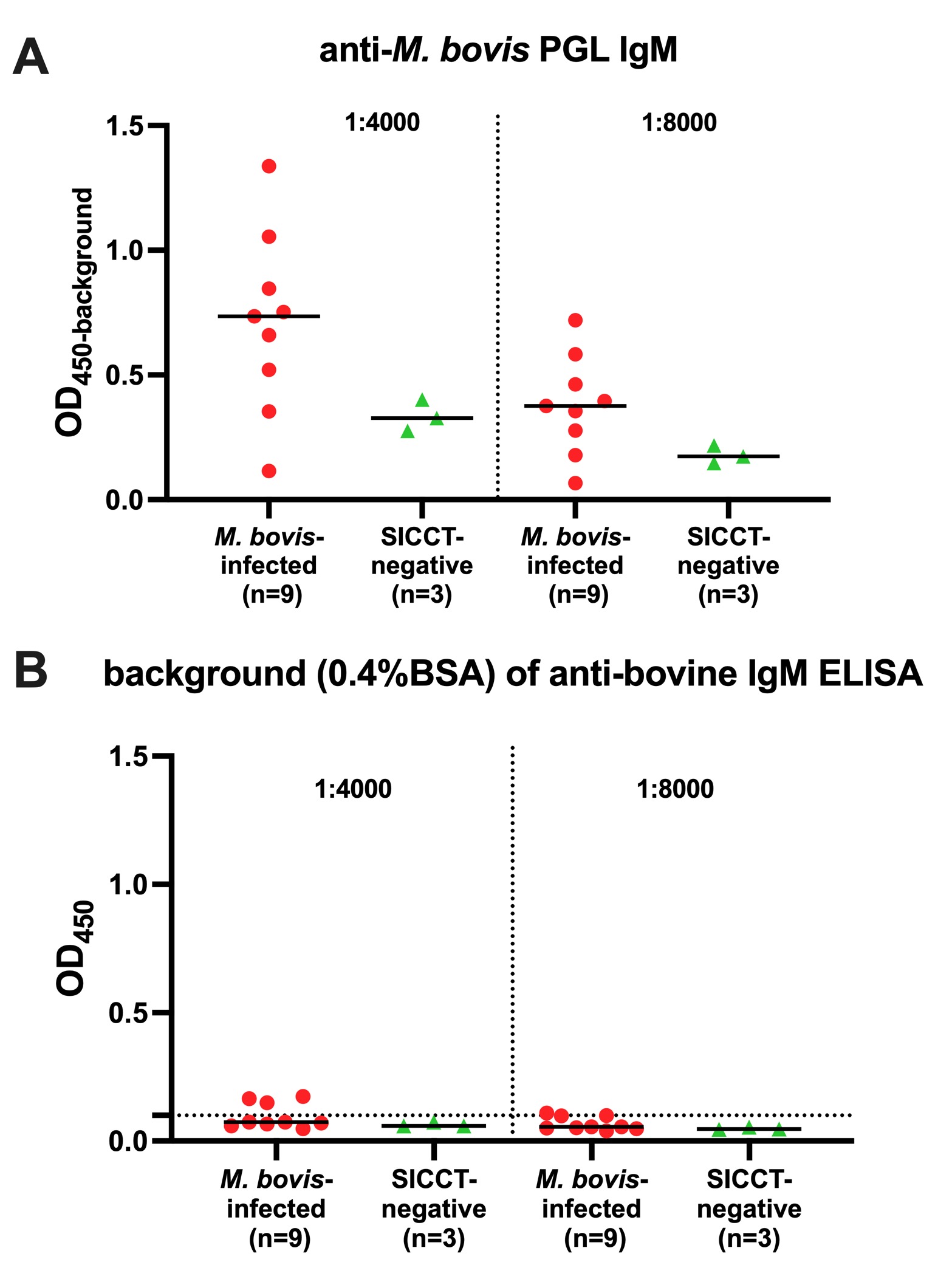


***Figure S4. Anti-M. bovis PGL IgM of different anti-bovine IgM antibody dilutions.*** *Nine anti-MPT83 IgG positive samples in M. bovis-infected (#15-23 in Table S1, red dots) and three SICCT-negative cattle (#15-18 in Table S2, green triangles) were tested in anti-M. bovis PGL ELISAs. M. bovis PGL (200 ng/well,* ***A****) and 0.4% BSA (****B****) were coated in 96-well microplates. Serum samples were diluted 1:400, and anti-bovine IgM antibody was diluted 1:4000 and 1:8000. Background values are displayed as optical densities at 450 nm (OD_450_); results of antibody levels are displayed as optical density at 450 nm corrected for background OD values (OD_450-background_). The median values of each group are indicated by horizontal lines.*


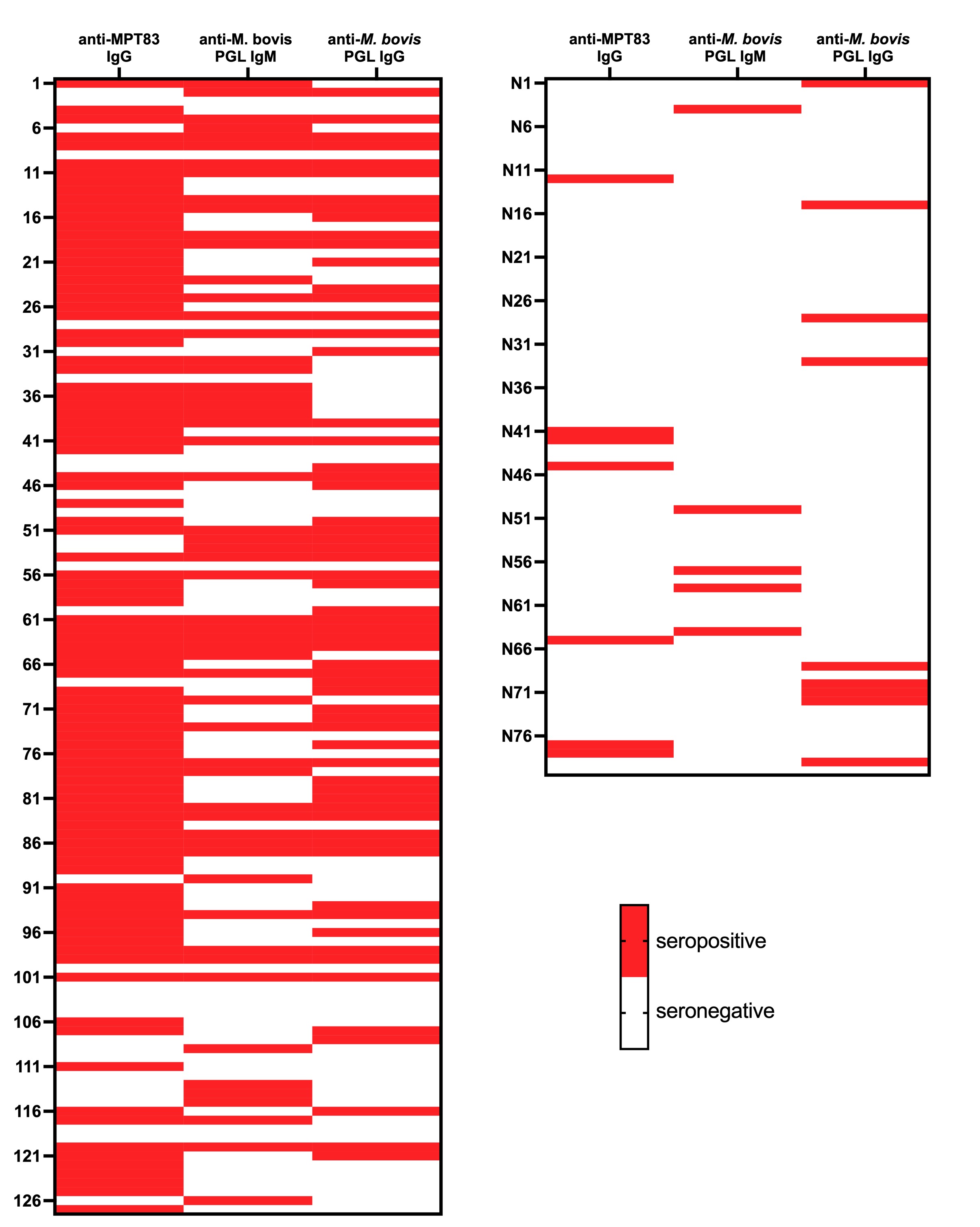


***Figure S5.*** ***Heatmap of ELISA results.*** *Anti-M. bovis PGL IgM and anti-MPT83 IgG ELISA results in cohorts A (n=127, left panel) and B (n=80, right panel) using cut-off values shown in* ***Fig. S3****,* ***Fig. 2*** *and* ***Tab. 1****; values above the cut-off for each biomarker are considered seropositive (marked in red).*


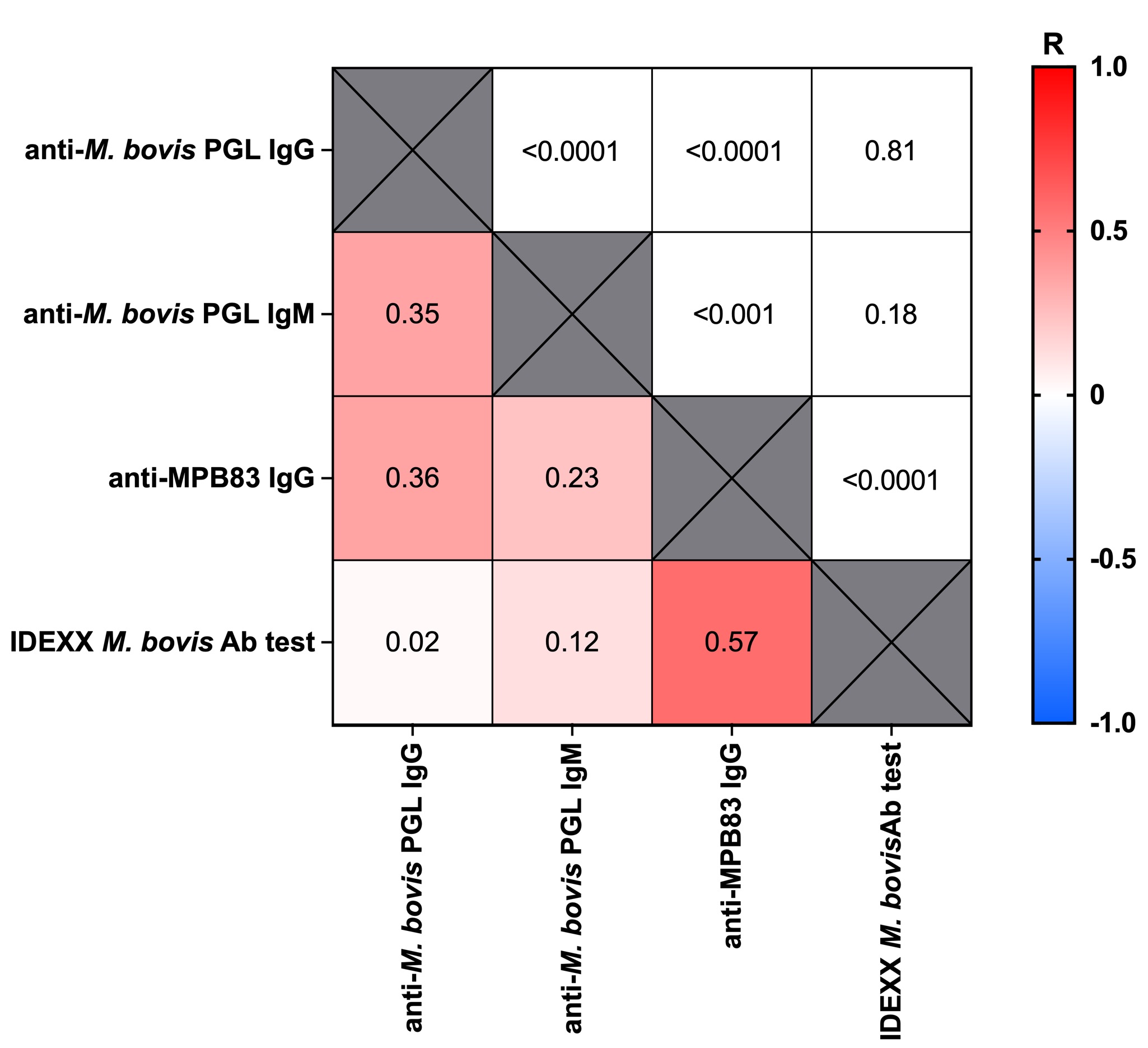


***Figure S6: Correlation between antibody levels.*** *Serum samples of M. bovis infected cattle (n=127) were assessed by anti-M. bovis PGL IgM/-IgG ELISA, anti-MPB83 IgG by ELISA, and IDEXX M. bovis Ab test. R values, the Spearman correlation coefficients, are depicted in bottom left corner of the heatmap. The corresponding p-values, indicating the significance level of the observed correlation between the different tests, are shown in upper right corner in white cells.*

Reference:

1. van Dijk, J.H.M., G.A. van der Marel, and J.D.C. Codée, *Developments in the Synthesis of Mycobacterial Phenolic Glycolipids.* Chem Rec, 2021. **21**(11): p. 3295-3312.

2. Barroso, S., et al., *Total Synthesis of the Triglycosyl Phenolic Glycolipid PGL-tb1 from Mycobacterium tuberculosis.* Angewandte Chemie International Edition, 2012. **51**(47): p. 11774-11777.

3. Barroso, S., et al., *Total Synthesis of the Phenolic Glycolipid Mycoside B and the Glycosylated p-Hydroxybenzoic Acid Methyl Ester HBAD-I, Virulence Markers of Mycobacterium tuberculosis.* European Journal of Organic Chemistry, 2013. **2013**(21): p. 4642-4654.
